# Insulin detemir in a twice daily insulin regimen versus a three times daily insulin regimen in the treatment of type 1 diabetes in children: A pilot randomized controlled trial

**DOI:** 10.1186/1687-9856-2011-15

**Published:** 2011-11-08

**Authors:** Josephine Ho, Carol Huang, Alberto Nettel-Aguirre, Danièle Pacaud

**Affiliations:** 1 Department of Pediatrics, University of Calgary, Calgary, T3B 6A8, Canada; 2 Department of Community Health Sciences, University of Calgary, Calgary, T3B 6A8 Canada

**Keywords:** type 1 diabetes, glycemic control, pediatric, long acting insulin analogue

## Abstract

**Background:**

Children with type 1 diabetes (DM1) often use three daily (TID) injections with intermediate acting insulin at breakfast and bedtime, and rapid acting insulin at breakfast and dinner. Substituting the evening intermediate acting insulin with a long acting insulin analogue (LAIA) at dinner in a twice daily (BID) injection regimen may be as effective as a TID regimen. The objective of this pilot study was to compare HbA1c in children with DM1 using a BID regimen with a LAIA at dinner (intervention) to those using a standard TID regimen (control) over 6 months.

**Methods:**

Randomized controlled trial with main outcome measure being HbA1c at 0, 3 and 6 months. Secondary outcomes were frequency of adverse events (hypoglycemia, diabetic ketoacidosis, weight gain) and scores on the Diabetes Quality of Life Measure for Youth (DQOLY).

**Results:**

18 subjects (10 control, 8 intervention). Mean years (standard deviations) for control and intervention respectively were: age at diagnosis of DM1 6.31 (2.91) vs 7.76 (3.22), duration of DM1 5.96 (4.95) vs 3.76 (3.37). No significant differences were seen in the mean HbA1c between control and intervention at 0 months [8.48(0.86) vs 8.57(1.13)], 3 months [8.47(0.50) vs 7.99(0.61)], or 6 months [8.42(0.63) vs 8.30(0.76)]. No significant differences were found between groups for frequency of adverse events or DQOLY.

**Conclusions:**

In this pilot study, incorporating LAIA in a BID regimen did not cause deterioration in HbA1c or increases in adverse events; suggesting that this may be a viable option for families where a more simplified insulin regimen would be beneficial and compliance may be improved.

**Trial registration:**

ClinicalTrials.gov: NCT00522210

## Background

Children with type 1 diabetes (DM1) require multiple daily injections of insulin to maintain good glycemic control. The Diabetes Control and Complications Trial (DCCT) has shown that intensive insulin treatment using at least three times daily (TID) injections achieves superior blood glucose control with decreased risk of long term complications than conventional insulin treatment using once daily or twice daily (BID) injections [1,2]. However, this study was done when long acting insulin analogues (LAIA) were not available which limited the types of insulin regimens and there are limited randomized controlled trials assessing analogue insulins in children. Multiple daily injection regimens are not consistently superior in children and other factors including patient support and team cohesion play large roles in glycemic control [3]. Many patients find it difficult to adhere to TID injections since it is an invasive and painful therapy, which results in frequent insulin omission. By replacing bedtime intermediate acting insulin with a LAIA at dinner, this would allow children with DM1 to maintain their glycemic control with only a BID insulin injection regimen.

The pharmacokinetic properties of the LAIA, detemir, have some potential benefits in the treatment of children with DM1 [4-8]. The longer duration of action would allow this long acting insulin analogue to be incorporated into a BID insulin regimen that could potentially offer equivalent glycemic control to a TID injection regimen. Intermediate and rapid acting insulin could still be given in the morning to avoid a lunchtime injection, while rapid acting and long acting insulin could be given at dinner to cover for the meal plus the background insulin required until the morning. Therefore, children only need to take insulin twice a day, which may result in greater compliance and improved quality of life. Satisfaction with diabetes treatment may also be improved because of more predictable glycemic control and less frequent adverse events. The risk of hypoglycemia may be decreased with detemir [4], because of the flat and protracted pharmacodynamic profile compared to the peak in insulin activity seen with intermediate acting insulins. Less frequent episodes of hypoglycemia may also result in less weight gain that can be seen in intensive insulin therapy.

The primary objective of this pilot, randomized controlled trial is to compare the glycemic control as measured by HbA1c in children with DM1 treated with a BID regimen of insulin using a LAIA overnight versus a TID insulin injection regimen with intermediate acting insulin. Secondary objectives included assessing the satisfaction with treatment of diabetes in each group using the Diabetes Quality of Life Measure for Youths (DQOLY) and determining the frequency of adverse events (severe hypoglycemia, nocturnal hypoglycemia, mild hypoglycemia, diabetic ketoacidosis, and change in body mass index (BMI)).

## Methods

### Study Design

The study was an open-labelled, randomized controlled trial design with two groups: control group (TID) and intervention group (BID). The trial was registered at *ClinicalTrials.gov Identifier: NCT00522210*.

### Subjects

Subjects were recruited from children with DM1 currently being followed at the Alberta Children's Hospital (Calgary, Alberta, Canada). Inclusion criteria were: children aged 6-17 years old, diagnosed with DM1 for at least 1 year and currently being treated with a TID regimen of insulin with rapid acting insulin and intermediate acting insulin. Exclusion criteria were: HbA1c ≥ 10% at enrolment, chronic underlying medical conditions that could affect glycemic control (examples: uncontrolled hypothyroidism, hyperthyroidism, celiac disease, etc.), current participants of other clinical trials, language or psychosocial barrier preventing the family from completing the study.

### Protocol

A sample of size of 65 subjects per group was initially calculated based on an estimated baseline HbA1c of 8.4% (SD 1.3%) in each group, a clinically acceptable difference of 10% (absolute difference of 0.84), a power of 90%, and a dropout rate of 20%. Therefore, if one assumed no drop-outs a sample size of 52 patients per arm would have been sufficient.

Subjects were randomized into the control group or intervention group using a computer generated randomization sequence (the sequence was generated in blocks to keep groups as balanced as possible and to help ensure allocation concealment). Subjects were stratified by age groups (6 to 10 years, and greater than 10 years old). Given the nature of the intervention, it was not possible to blind patients, their parents, and caregivers to the treatment allocation.

In the control group (ie. the TID regimen), subjects were asked to continue on their usual insulin regimen (intermediate acting insulin and rapid acting analogue at breakfast, rapid acting analogue at dinner and intermediate acting insulin at bedtime). In the intervention group (ie. the BID regimen), the subjects' usual bedtime dose of intermediate acting insulin was discontinued and replaced with a dose of insulin detemir at dinner time. The intermediate acting insulin used was neutral protamine hagedorn (either Novolin NPH or Humulin N depending on what the patient was currently using). The rapid acting analogues used were lispro insulin (Humalog) or aspart insulin (Novorapid) depending on what the patient was currently using. The detemir was not mixed with the rapid acting insulin and was given as a separate injection. The dose of detemir was approximately 50% of the total daily dose of insulin, with the remaining 50% being comprised of the subject's breakfast dose of intermediate acting insulin and rapid acting analogue and dinner rapid acting analogue. A run-in period of 1 month, with a minimum of a weekly phone contact, was used to facilitate the change in insulin regimen and dose finding for the intervention group and to optimize insulin doses in the control group. No changes were made to the subjects' usual diet and exercise routines.

**Figure 1:**
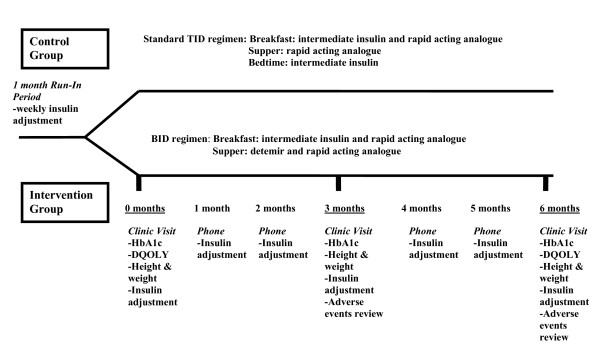
**Study design and subject follow up**.

Throughout the study, monthly phone contact for insulin adjustments was done for both groups. In addition, subjects were assessed in clinic at baseline, 3 months and 6 months (Figure 1). This included height, weight, HbA1c, current insulin doses, episodes of severe hypoglycemia (glucose less than 4 mmol/L associated with a decreased level of consciousness, seizure, or coma), reported nocturnal hypoglycemia, mild hypoglycemia (glucose less than 4 mmol/L where the patient is able to self treat) and diabetic ketoacidosis (hyperglycemia and ketonuria associated with a pH < 7.3 and/or bicarbonate level < 15 mmol/l).

The Diabetes Quality of Life Measure for Youths (DQOLY) was administered at baseline and again at 6 months. This questionnaire was initially used by the DCCT group and was later revised by Ingersoll et al [9]. It has been validated in youths aged 10-21 years. This instrument has three Likert scales including a 17 item diabetes life satisfaction scale (range of scores 17-85), 23 item disease impact scale (range of scores 23-115), and an 11 item disease related worries scale (range of scores 11-55) [9]. In this study, reverse scores were recorded for the impact and worries scales so that a higher score indicated a better quality of life. For the satisfaction scale, a higher score indicates higher satisfaction.

### Data Analysis

Baseline demographic and clinical variables are presented as means with standard deviations (SD) for numerical variables and as proportions for categorical variables. The 95% confidence interval for the difference between groups is presented too. At each time point, HbA1c between the two groups was compared using a two sample t-test and confidence interval for the difference is provided to help assess non-inferiority. The true expected difference in HbA1c between the control and treatment group was taken to be zero. Based on previous follow up data from the ACH Diabetes Clinic, the mean HbA1C is estimated at 8.4% with a standard deviation of 1.3 for each group. A 10% relative difference was considered a clinically acceptable difference. Therefore, the non-inferiority margin was set at 0.84.

### Ethical approval

The protocol, including subject information, informed consent, recruitment procedure, interventions and data collection has been approved by the Conjoint Health Research Ethics Board of the Faculty of Medicine, University of Calgary (Calgary, Alberta, Canada) in accordance with the Declaration of Helsinki and Tri-Council Guidelines.

## Results

Table 1 shows the enrolment characteristics of the subjects. There were no significant differences between the groups at baseline. In total, 18 subjects were enrolled (10 control, 8 intervention). The mean age at diagnosis of DM1 was 6.31 years (SD 2.91) for control and 7.76 years (SD 3.22) for intervention. The mean duration of DM1 was 5.96 years (SD 4.95) for control and 3.76 years (SD 3.37) for intervention.

**Table 1:** Enrolment characteristics of subjects.

Characteristic	Control (TID) N = 10	Intervention (BID) N = 8	Difference between groups (95% confidence interval for the difference)
Gender (Female/Male)	4/6 (40% female)	4/4 (50% female)	10% (-50.02% - 33.36%)

Age groups (children < 10 years old/children ≥ 10 years old)	3/7 (30% children < 10 years old)	3/5 (37.5% children < 10 years old)	7.5% (-47.08% - 33.74%)

Age at study enrolment (years)	12.26 (3.40)	11.52 (2.08)	-0.74 (-3.52 - 2.03)

Age at type 1 diabetes diagnosis (years)	6.31 (2.91)	7.76 (3.22)	1.45 (-1.68 - 4.58)

Duration of diabetes (years)	5.96 (4.95)	3.76 (3.37)	-2.20 (-6.37 - 1.98)

Last HbA1c prior to enrolment (%)	8.54 (0.70)	8.70 (0.58)	0.16 (-0.48 - 0.80)

Insulin dose (units/kg/day)	1.02 (0.40)	0.94 (0.24)	-0.08 (-0.41 - 0.25)

Body Mass Index (kg/m^2^)	20.62 (4.07)	20.99 (3.60)	0.38 (-3.46 - 4.22)

**No significant differences were found between the groups at baseline**. Table shows Number (%) or Mean (Standard deviation). Difference is for intervention minus control.

There were no significant differences in the mean HbA1c between control and intervention groups at 0 months [8.48 (SD 0.86) vs 8.57 (SD 1.13)], 3 months [8.47 (SD 0.50) vs 7.99 (SD 0.61)], or 6 months [8.42 (SD 0.63) vs 8.30 (SD 0.76)] (Table 2). Adverse events, such as DKA and reported hypoglycemic episodes, were similar in frequency in the control and intervention groups. There were no significant differences in body mass index or quality of life scales between groups (Table 2). The width of the confidence intervals for each of the outcome measures was large, likely due to the small sample size. However, they do indicate that the intervention was non-inferior when compared to the control group.

**Table 2:** Results

		Baseline			3 Months			6 Months	
	**Control**	**Intervention**	**Difference** **(95% confidence interval)**	**Control**	**Intervention**	**Difference** **(95% confidence interval)**	**Control**	**Intervention**	**Difference** **(95% confidence interval)**

HbA1c %	8.48 (0.86)	8.57 (1.13)	0.095 (-0.95 - 1.14)	8.47 (0.50)	7.99 (0.61)	-0.48 (-1.06 - 0.095)	8.42 (0.63)	8.30 (0.76)	-0.12 (-0.84 - 0.60)

Body Mass Index (kg/m2)	20.62 (4.07)	20.99 (3.60)	0.38 (-3.46 - 4.22)	21.81 (6.40)	22.21 (3.48)	0.39 (-4.67 - 5.46)	21.04 (3.87)	21.75 (3.58)	0.71 (-3.03 - 4.45)

DKA (episodes in last 3 months)	0	0	0	0	0	0	0	0	0

Severe Hypoglycemia (episodes in last 3 months)	0.10 (0.32)	0	-0.10 (-0.33 - 0.13)	0.30 (0.67)	0	-0.30 (-0.78 - 0.18)	0.1 (0.32)	0	-0.10 (-0.33 - 0.13)

Mild/Moderate Hypoglycemia #/week	2.11 (1.40)	2.16 (1.65)	0.046 (-1.53 - 1.62)	2.17 (1.76)	1.87 (1.34)	-0.30 (-1.85 - 1.25)	2.12 (1.31)	2.54 (1.36)	0.41 (-0.94 - 1.77)

Nocturnal Hypoglycemia #/week	0.15 (0.34)	0.62 (1.03)	0.47 (-0.39 - 1.34)	0.12 (0.32)	0.72 (0.91)	0.59 (-0.18 - 1.36)	0.26 (0.57)	0.50 (0.72)	0.24 (-0.43 - 0.91)

QOL Impact	83.78 (7.38)	90.37 (8.14)	6.60 (-1.50 - 14.70)	---	---	---	83.70 (17.57)	91.00 (8.45)	7.30 (-6.28 - 20.88)

QOL Worries	41.62 (6.30)	42.00 (5.76)	0.37 (-6.10 - 6.85)	---	---	---	37.90 (8.55)	39.37 (7.29)	1.47 (-6.45 - 9.40)

QOL Satisfaction	67.33 (8.37)	67.00 (10.42)	-0.33 (-10.28 - 9.61)	---	---	---	65.00 (12.62)	72.50 (8.37)	7.50 (-3.06 - 18.06)

Comparison of glycemic control, adverse events and quality of life scores between the control and intervention groups. **No significant differences were detected between the groups at baseline, 3 months or 6 months**. Dashed line indicates data was not collected at that time point. Control (TID) N = 10. Intervention (BID) N = 8. Difference is for intervention minus control. Table shows Mean (Standard Deviation).

## Discussion

Currently, a standard TID injection regimen with intermediate acting insulin at breakfast and bedtime, and rapid acting insulin at breakfast and dinner is often used in children. Families that opt for more intensive therapy can choose a continuous subcutaneous insulin infusion or a basal bolus regimen. However, these regimens are costly and require a significant amount of skill and effort from the family. In addition, patient compliance with multiple invasive and painful injections can be an issue when using multiple injections. Another challenge with exogenous insulin administration is hypoglycemia. While the results of the DCCT clearly demonstrated the importance of maintaining a near normal glucose level, the major adverse event reported in the intensive insulin treated subset of patients aged 13-17 years was a nearly three fold increase in severe hypoglycemic events [1,2]. The development of new LAIA offers the opportunity to simplify insulin regimens while achieving similar glycemic control.

Detemir is a LAIA that has prolonged insulin absorption with less intra-patient variability in peak insulin activity as well as very minimal peak activity, in comparison with intermediate acting insulin [10,11]. The pharmacokinetic properties of detemir have been studied in patients with DM1 using a euglycemic glucose clamp technique [5,12]. In comparison to neutral protamine hagedorn (NPH) insulin, detemir resulted in a more stable serum concentration of insulin without the peak seen in NPH [5,12]. In addition, there were less fluctuations in the glucose infusion rates required with detemir in steady state compared to NPH in steady state [5,12]. Detemir has been shown to have a consistent pharmacokinetic profile in children, adolescents, and adults with DM1[7,8].

Recently, a retrospective study by Cengiz et al [13] analyzed the same BID regimen (NPH and rapid acting insulin analogue at breakfast with insulin detemir and rapid acting insulin analogue at dinner) in children with new onset DM1 as an option prior to initiation of insulin pump therapy. They found that by 12 months after diagnosis of DM1, 49 of the patients had changed to pump therapy with a median HbA1c of 6.9% while 59 remained on the BID injection regimen with a median HbA1c of 7.2% [13]. The authors concluded that this BID regimen was effective in children with new onset diabetes and had similar glycemic control [13]. The findings of this retrospective study are consistent with our results, as we also did not find a difference in glycemic control for patients on TID versus the BID regimen. In contrast to Cengiz et al's study [13], our randomized control trial was aimed at assessing the effectiveness of a BID regimen in children with DM1 greater than 12 months as an option for families where intensive diabetes therapy was not feasible and improved quality of life could be achieved by simplifying the diabetes regimen.

HbA1c changes when using insulin detemir has been studied in children and adolescents with conflicting results [14-17]. Braun et al [16] reported improved HbA1c and fewer severe hypoglycemic episodes in a chart review study of children who were switched from evening NPH insulin to detemir. Interestingly, in this study a subset of children under 12 years of age were treated with a BID regimen and showed an improvement in HbA1c from 8.3% to 7.6% after changing from evening NPH to detemir [16]. Dundar et al [14] reported an improvement in HbA1c in children who changed from a basal bolus regimen with NPH to either glargine or detemir. This study was limited by the fact that it was retrospective and was small with only 15 patients in the detemir treated group [14]. In a large, prospective, 26 week, randomized study of 347 children, Robertson et al [15] reported no difference in HbA1c in children changed to basal bolus therapy with either NPH or detemir compared to pre-basal bolus therapy. Although HbA1c was not better in the detemir group, the risk of nocturnal hypoglycemia was 26% lower [15]. Kurtoglu et al [17] retrospectively assessed children that were initially on basal bolus regimens with NPH or glargine then switched to detemir. After 12 weeks of using detemir, HbA1c was improved and the frequency of hypoglycemic episodes was decreased [17]. Although we did not see a similar improvement in HbA1c in our patients using detemir, these studies examined detemir in basal bolus regimens rather than the BID regimen used in our study. It is also reassuring that our study did not find a worsening of glycemic control despite simplifying the insulin regimen.

Hypoglycemia can be a side effect of intensive insulin therapy. Several studies have demonstrated that detemir is associated with a decreased frequency of hypoglycemia since it does not have a peak activity [4,18]. In a randomized, open, cross-over trial [18], detemir has been compared to NPH insulin in a basal bolus regimen in adults with DM1 and has been found to be as effective as NPH in maintaining glycemic control. Fewer patients experienced hypoglycemia with detemir compared to NPH [18]. Vague et al [4] compared detemir and NPH in 448 adult patients with DM1 in a basal-bolus regimen using twice daily detemir or NPH for basal coverage and rapid acting insulin with each meal. In their detemir group, more predictable glycemic control was seen during night-time plasma glucose monitoring using an intravenous line [4]. A significant reduction in the frequency of hypoglycemia as well as weight gain was also seen in their detemir group [4]. Interestingly, there was no difference in the HbA1c between the two groups after 6 months [4]. In our study, no differences in reported episodes of hypoglycemia were seen between groups.

Weight gain can be a concern when children are on intensified insulin regimens or have frequent hypoglycemia. Home et al [6] conducted a 16 week, randomized control trial of 408 patients with type 1 diabetes. HbA1c improved by 0.18% in the group using insulin detemir as the basal insulin compared to NPH insulin [6]. In addition, there was a decreased frequency of hypoglycemia and no weight gain in the group using insulin detemir compared to the NPH group which did have some weight gain [6]. Although the insulin regimen used in our study was not a basal bolus one, we did not find any significant changes in BMI between the groups.

Diabetic ketoacidosis (DKA) was not seen in either the control or intervention group. This is not necessarily surprising given the short duration of this study and small sample size. Karges et al [19], compared the incidence of DKA in a cohort of 10 682 children and adolescents with DM1 being treated with either NPH insulin or a LAIA. They found that the incidence of DKA was significantly higher in patients using glargine or detemir compared to those using NPH [19]. However, all of the patients studied were on at least three or more insulin injections per day while our patients using detemir were on a BID regimen.

Quality of life measures were not significantly different between the two groups using the DQOLY. A limitation of using the DQOLY was that this questionnaire has only been validated in youths aged 10-21 years [9]. Our study included children aged 6 years and older. However, only 1 subject was 7 years old and 5 subjects were 8 to 9 years old at enrolment and the DQOLY was the most practical and accessible measure to use at the time of this study.

At the time the study was conducted, it was recommended that families could continue mixing the intermediate insulin and rapid insulin analogue in one syringe in the morning. However, at supper time the LAIA and rapid insulin were to be given in two separate injections. Recently, Nguyen et al [20] published a study of 14 children with type 1 diabetes who underwent continuous glucose monitoring and found that mixing insulin detemir with aspart had equivalent effects on blood glucose when compared with giving them as separate injections. The ability to mix insulin detemir with aspart at supper time would again simplify the regimen for families and potentially have a greater impact on satisfaction and compliance.

A significant limitation of this study is the small sample size. Interestingly, recruitment was difficult for this study. Once families received a description of the alternate BID insulin regimen compared to the traditional TID regimen, many did not want to risk being randomized to the control group. The theoretical benefits of decreased nocturnal hypoglycemia and twice daily insulin injection times was very attractive to families; particularly those that struggle with compliance.

## Conclusions

The results of this pilot study demonstrate that using a BID insulin regimen incorporating a LAIA allows for maintenance of glycemic control despite a less intensive injection regimen. Ideally, a basal bolus regimen with multiple daily injections or an insulin pump would mimic physiologic insulin secretion most closely, but practically this is often difficult to achieve in young children who are dependent on a responsible adult to be available for injections.

Simplifying to BID insulin regimens incorporating LAIA may be possible with no increase in adverse events and comparable HbA1c compared to standard TID regimens used in children, although larger clinical studies would be required to confirm this finding. Although no significant improvements were seen in DQOLY and nocturnal hypoglycemia, it is important that HbA1c remained stable, and suggests that this regimen is a viable option for families.

## Competing interests

The authors declare that they have no competing interests.

## Authors' contributions

JH designed the study; collected, analyzed and interpreted the data; and drafted the manuscript. CH, AN and DP significantly contributed in the study design, data analysis, data interpretation and revising of the manuscript. All authors read and approved the final manuscript.
